# An updated meta-analysis of the primed goal-organizational behaviour relationship

**DOI:** 10.1098/rsos.221494

**Published:** 2023-04-12

**Authors:** Gary P. Latham, Xiao Chen, Ronald F. Piccolo, Guy Itzchakov

**Affiliations:** ^1^ Rotman School of Management, University of Toronto, 105 St. George Street, Toronto, ON Canada, M5S 2E8; ^2^ Faculty of Business, University of Prince Edward Island, 550 University Ave, Charlottetown Prince Edward Island, PE, Canada C1A 4P3; ^3^ College of Business, University of Central Florida, 12744 Pegasus Dr., Orlando, FL 32816, USA; ^4^ Faculty of Social Welfare and Health Sciences, University of Haifa, 199 Aba Khoushy Ave., Mount Carmel, Haifa, Israel 3498838

**Keywords:** priming, organizational behaviour, goal-setting theory, meta-analysis

## Abstract

Environmental cues (e.g. achievement-related words and pictures) can prime/activate, in the absence of awareness, a mental representation of importance stored in memory. Chen *et al*.'s 2021 *Applied Psychology: An International Review* **70**, 216–253. (doi:10.1111/apps.12239) meta-analysis revealed a moderate, significant overall effect for the goal priming-organizational behaviour relationship, with three moderators identified: context-specific versus a general prime, prime modality (i.e. visual versus linguistic) and experimental setting (field versus laboratory). An independent researcher found that their finding was negligibly affected by a publication bias. Shanks & Vadillo (2021), *Royal Society Open Science* **8**, 210544. (doi:10.1098/rsos.210544) (field: *k* = 13, *N* = 683, *d* = 0.64), questioned Chen *et al*.'s conclusion regarding the effect size found in field studies (field: *k* = 8, *N* = 357, *d* = 0.68). In this paper, we discussed Shanks & Vadillo's selection of additional field experiments that led to their conclusion of a publication bias. We updated Chen *et al*.'s meta-analysis to include relevant studies conducted since that study's publication. The present meta-analysis reproduced the original findings in Chen *et al*. (field: *k* = 11, *N* = 534, *d* = 0.67). The updated findings are consistent with: (i) laboratory findings, (ii) the findings obtained in field experiments on consciously set goals and (iii) goal setting theory (Latham & Locke, 2018 In *Handbook of industrial, work & organizational Psychology*, vol. **1** (eds D Ones, N Anderson, C Viswesvaran, H Sinangil), pp. 103–124).

## 1. Introduction

Shanks *et al.* (e.g. [1–5]) have a long history of arguing against the findings from meta-analyses of priming experiments. Continuing with this trend, Shanks & Vadillo [6] critiqued our meta-analysis of goal priming experiments in organizational psychology [7]. In this article, we briefly describe our previous meta-analysis, comment on Shanks and Vadillo's criticism of one of our three sub-analyses, and present an updated meta-analysis of the primed goal-organizational behaviour relationship.

Several factors differentiate Chen *et al*.'s [7] meta-analysis from previous meta-analyses (e.g. [8]). First, the overall dependent variable was specific to organizational-related behaviour (e.g. brainstorming). Second, the experiments included in the meta-analysis were conducted in field settings in addition to a laboratory. Third, the meta-analysis was preceded by an enumerative literature review. Fourth, and arguably most importantly, the majority of the laboratory and field experiments on goal priming were conducted within a theoretical framework, namely, goal setting theory [9–11].

In this article, we strive to ‘stay above the weeds’, such as what does or does not constitute a field experiment, as we suspect the inter-observer reliability on this issue in organizational psychology is relatively high. Instead, we focus on the main findings of Shanks & Vadillo's [6] re-analysis of our data.

First, and most importantly, we note that Shanks & Vadillo acknowledged that they reproduced our finding that there is a goal priming relationship with organizational behaviour ([6]; personal communication, 11 May 2020). Consequently, Shanks & Vadillo focused their critique on one of three sub-analyses, namely, the finding that the effect size for goal priming experiments conducted in the field, contrary to our hypothesis [7], is greater than that for goal priming experiments that were conducted in the laboratory (‘Hypothesis 5: *Primed goal effects in laboratory settings are replicable in field settings; however, the effect sizes in the laboratory are greater than in the field*’, p. 230). They then devoted their paper to showing that the eight field experiments in our sub-analysis are underpowered, susceptible to a publication bias, and hence the effect sizes between them and the laboratory experiments are no longer meaningful when correcting for it.

In Chen *et al*.'s [12] rejoinder to three commentaries [13–15] on our paper [7], we presented the results of an analysis for publication bias of organizational behaviour-related experiments on the primed goal-organizational behaviour relationship. To increase objectivity, we took the rare step in the field of psychology of requesting a researcher who does not conduct experiments on goal priming and hence is neither a skeptic nor an advocate of primed goal findings,^1^ to determine whether the overall effect size we reported reflects a publication bias. Her analysis showed a negligible publication bias that did not change the conclusion. Unlike Shanks & Vadillo, she did not examine the possibility of a publication bias in the sub-analysis of the effect sizes of field versus laboratory experiments. This is because the number of field experiments in organizational psychology on goal priming, a research domain in its early stage, is so low that the possibility of a publication bias is self-evident.

Shanks & Vadillo also reached another readily observable conclusion, namely, that each field experiment in our analysis is underpowered. It is exceedingly difficult, if not impossible, for an experimenter to persuade an organization's senior management to hire additional employees to increase the statistical power of a psychological experiment. Among the benefits of meta-analyses, however, is an aggregation of the data so as to overcome the limitation of underpowered experiments [16].^2^ Moreover, it is instructive to note that the findings of the goal priming field experiments are consistent with the laboratory findings reported by Chen *et al*. [7]. Furthermore, the findings of the field experiments on primed goals are consistent with those obtained in field experiments on consciously set goals [9]. Most importantly, the findings of field experiments involving primed goals, as is the case with field experiments involving consciously set goals, are consistent with goal setting theory [9,10,18].

The first organizational psychology experiment on goal priming [19] was conducted in a laboratory where the dependent variable was brainstorming uses for a coat hanger. Brainstorming is a commonly used task in organizations. Subsequently, two field experiments on goal priming were conducted [20].

Shanks & Vadillo [6] argued that the first of two field experiments conducted by Shantz & Latham [20] involving brainstorming should not have been coded as a field experiment in our sub-analysis. Hence, they deleted it in their sub-analysis of our field data. That first experiment was included in our analysis [7] because all the participants were white-collar employees coming to work through Union Station. Union Station is to Toronto what Grand Central Station is to New York City.

The dependent variable in that field experiment, brainstorming uses for a coat hanger, was used to determine whether the results obtained by Stajkovic *et al*. [19] with college students could be replicated. When that field experiment was conducted, the present first author, like Shanks, was a skeptic of goal priming findings in social psychology [21]. Hence, replication was considered necessary before attempting to persuade an organization's decision makers to permit a field experiment to determine whether their employees' job performance can be increased by priming a performance goal.^3^

Shanks & Vadillo [6] correctly pointed to the fact that two field experiments ([23], experiment 4; [24], experiment 3) conducted by the first and fourth authors of the present paper were not included in Chen *et al*.'s [7] meta-analysis. This is because those two experiments had yet to be conducted when we were conducting the meta-analysis. However, it is noteworthy that the results of both those experiments revealed a significant positive effect of a primed goal on performance. Nevertheless, we excluded Itzchakov & Latham's [23] experiment 4 in our up-dated meta-analysis because the experimental design was a 2 × 2 × 2 between-within participant design, with prime (neutral/effective) and feedback (no/yes) as between-participant factors, and time (performance before a shift/performance during a shift) as a within factor.

In short, in our original meta-analysis, we were seeking to estimate the between-participants effect of a primed goal relative to a no-prime control condition. Hence, we did not include any within-participant effects of a primed goal relative to a no-prime control condition because we do not believe that this design allows for a direct comparison between a primed goal versus a no prime control condition. Shanks & Vadillo used the means and s.d. of the pre-and-post performance measures and compared the primed goal condition to the control condition. Whereas their analysis sought to estimate *within*-participant effects of a priming condition relative to a control, a within-participants effect was not the focus of our meta-analysis, nor was it hypothesized as a potential reflection of goal setting theory.

In addition, the first of three of Bipp *et al*.'s [25] experiments was not included in our original sub-analysis as a field experiment because of a coding error. Arguably, academic grades are consequential for students, as are performance appraisals for employees. And an academic institution is as much an organization as those in the private and public sectors. Hence, we included Bipp *et al*.'s first experiment, a field experiment involving students and their grades, in our updated analysis reported in this paper, as did Shanks & Vadillo [6].

A field experiment conducted by Stajkovic *et al*. [26] was not included in our initial meta-analysis because we were unable to obtain the necessary descriptive statistics from the first author (Stajkovic, email, 4 June 2018). Their online published paper did not report performance means and s.d. as of our cut-off date (5 November 2018) for including experiments in our meta-analysis. These data were subsequently reported in their journal publication. Thus this experiment, included in the Shanks & Vadillo meta-analysis, is now included in our updated analysis.

It is noteworthy that Shanks & Vadillo [6], after providing a detailed report on their exhaustive review of the literature, only found one experiment (effect size *k* = 2: runner versus control; canvasser versus control) that we missed, namely a field experiment conducted by Lenoir & Matthews [27] involving door to door election canvassing. This experiment appeared online (3 October 2019) after we had submitted our findings for publication. But, the canvassers in that study were not employees who work in an organizational setting. They were unpaid volunteers for a mayoral campaign. Thus, we did not include the results of that experiment in our updated analysis even though the data provided a conceptual replication of Shantz & Latham's ([20], experiment 2)^4^. We do not consider door-to-door canvassing by unpaid volunteers for a mayoral campaign to fall under the rubric of organizational behaviour. In fact, most research on canvassing has been published in the field of political psychology (e.g. [30–32]). The number of doors knocked on does not equate with job performance. For example, Canvasser A who knocked on 50 doors and persuaded 30 people to vote for a preferred candidate is a higher performer than Canvasser B, who knocked on 100 doors yet only persuaded 20 people to vote for the preferred candidate. Lenoir & Matthews did not take this issue into account. Thus, our updated meta-analysis excludes this experiment (as noted above, *k* = 2) that Shanks & Vadillo included in their analysis.

In summary, Shanks & Vadillo [6] excluded one experiment that we included in our updated analysis, namely, the first of two experiments conducted by Shantz & Latham [20], and we excluded one field experiment that they included in their meta-analysis (i.e. [27]).

## 2. Updated meta-analysis

### 2.1. Literature search

In the current study, we updated the estimated effect sizes of the meta-analysis reported in Chen *et al*. [7]. To do so, we started with the dataset used by Chen *et al*. that included 23 articles that yielded 40 independent effect sizes (*n* = 3179, *k* = 40). Using the same criteria and methodology, we searched *PsychInfo* for peer-reviewed experiments on the primed goal effects published between June 2020 and November 2021^5^. Specifically, we used the following key words: prime, primed goal, goal priming, subconscious goal, non-conscious goal, unconscious goal and performance. In addition, we included ‘goal setting theory’ in the ‘TX All Text’ search. This search yielded an additional 32 articles including the Itzchakov & Latham experiment [24].

Consistent with Chen *et al*. [7], an effect size was included in the current meta-analysis if it met four criteria: (i) the presence of a prime for a performance goal for achievement, (ii) dependent variables that are relevant to organizational behaviour (e.g. task and job performance), (iii) an experimental design with a control (no prime) condition and (iv) the availability of statistics needed to calculate an effect size (e.g. s.d.). Given these criteria, three additional experiments were included in the updated dataset [33–35].

As in Chen *et al*. [7], we converted results from the primary experiments in our database to standardized mean difference scores (i.e. Cohen's *d;* [36]). Updated point estimates of primed goal effects were calculated using the meta-analytic methods recommended by Hunter & Schmidt [17], which as noted earlier, ‘correct not only for sampling error (an unsystematic artifact) but also for other, systematic artifacts such as measurement error, range restriction or enhancement, dichotomization of measures, and so forth’ (p. 339). In our updated dataset, no corrections were made for range restriction or measurement error because all studies in our analysis were experiments.

## 3. Results

As reported in table 1, the overall effect of a primed goal for achievement on performance and need for achievement was significant and positive (*d* = 0.44, *p* < 0.05). In Chen *et al*. [7], the overall effect size was similar (*d* = 0.45). As reported in the original study, a primed goal for achievement had significant effects on performance (*d* = 0.42, *p* < 0.03) and the need for achievement (*d* = 0.69, *p* < 0.05). The effect sizes obtained by Chen *et al*. were *d* = 0.44 and 0.67, respectively. Thus, the inclusion of additional articles in the present analysis did not materially change the estimated effect sizes of a primed goal for achievement.

**Table 1.** Meta-analytic results of primed goal for achievement. Note. *n* = sample size. *k* = number of effects. *d* = Cohen's *d*. 95% CI = 95% confidence interval.

	*n*	*k*	*d*	95% CI
overall effect	3449	43	0.44	(0.36, 0.51)

dependent variables	*n*	*k*	*d*	95% CI
performance	3,106	38	0.42	(0.35, 0.50)
need for achievement (*n Ach*)	322	6	0.69	(0.53, 0.85)

In table 2, we report the results of our updated moderator analyses. As in Chen *et al*. [7], we calculated separate meta-analytic point estimates for subsets of our dataset based on moderator variable categories. We then conducted a *Z*-test to determine if the differences were statistically significant [37]. All three of the additional studies were laboratory experiments with an assessment of outcomes within a few minutes following the prime. Selvì & Sümer [34] used a visual modality to prime achievement. Both Sergent [35] & Keller [33] used a linguistic modality as a prime.

**Table 2.** Moderator analysis of results of primed goals for achievement. Note. *n* = sample size. *k* = number of effects. *d* = Cohen's *d*. 95% CI = 95% confidence interval. * *p* < 0.05. *^F^ F*-statistic from single factor *ANOVA*.

moderators					
	*n*	*k*	*d*	95% CI	*Z*
prime specificity					
context-specific	152	3	0.91	(0.80, 1.02)	10.94*
general	3667	45	0.45	(0.37, 0.53)	
modality					
visual	1821	22	0.56	(0.45, 0.66)	3.19*
linguistic	1916	24	0.38	(0.27, 0.48)	
time lag					
seconds	582	5	0.38	(0.14, 0.62)	1.61*^F^*
minutes	2592	30	0.46	(0.37, 0.55)	
hours	574	11	0.57	(0.38, 0.76)	
days	71	2	0.81	(0.53, 1.08)	
experimental setting					
field	534	11	0.67	(0.49, 0.85)	4.75*
laboratory	3285	37	0.44	(0.35, 0.52)	

Overall, the pattern of effect sizes in our updated analysis is consistent with that reported by Chen *et al*. [7]. The point estimate of a context-specific primed goal in this analysis (*d* = 0.91, *p* < 0.05) was unaffected by the additional studies and remained significantly higher (*Z* = 10.94, *p* < 0.05) than the new point estimate of a general primed goal (*d* = 0.45, *p* < 0.05). The point estimate for a general primed goal reported by Chen *et al*. was *d* = 0.42 (*p* < 0.05).

The new point estimate of a visual prime (*d* = 0.56, *p* < 0.05) is significantly higher (*Z* = 3.19, *p* < 0.05) than the new point estimate of a linguistic prime (*d* = 0.38, *p* < 0.05). These results are consistent with those reported by Chen *et al*. [7].

As in our original study, we were interested in the extent to which priming effects vary over time. We separated studies, depending on the time lag, between the priming effect and the assessment of the outcomes (e.g. seconds, minutes, hours, days). Point estimates for seconds (*d* = 0.38, *p* = 0.05) and days (*d* = 0.81, *p* < 0.01) were unchanged. The updated effect sizes for time lag in minutes and hours changed slightly from *d* = 0.45 (*p* < 0.05) to *d* = 0.46 (*p* < 0.05), and from *d* = 0.47 (*p* < 0.05) to *d* = 0.57 (*p* < 0.05), respectively. As in our original study, differences in effect sizes across the time categories are not statistically significant.

Lastly, similar to Chen *et al*. [7], the point estimate for the priming effect in a field experiment (*d* = 0.67, *p* < 0.05) is significantly higher (*Z* = 4.75, *p* < 0.05) than the new point estimate of a laboratory prime (*d* = 0.44, *p* < 0.05). This finding is not only consistent with that reported by Chen *et al*. [7], it is contrary to our hypothesized effect in the original study.

## 4. Discussion

The critical tone in which Shanks & Vadillo wrote their critique of a sub-analysis reported by Chen *et al*. [7] suggests a number of ironies. First, their implicit, if not explicit, message is a lack of transparency on our part. This is ironic in that, as previously noted, Shanks & Vadillo were able to reproduce the overall effect that Chen *et al*. obtained regarding the primed goal-performance relationship after we provided them with all the information they requested, followed by their exhaustive review of the literature on this subject matter.

Second, there is an undertone of skepticism as to why several field experiments conducted by Latham and colleagues were not included in the Chen *et al*. sub-analysis comparing effect sizes of laboratory versus field experiments. The implication is that Chen *et al*. were biased in their inclusion/exclusion of field experiments conducted with employees. This implication makes little sense in that there is no logical reason for us to exclude experiments that show a significant causal relationship between a primed goal and performance. These field experiments conducted by one or more of the present authors, included in the Shanks & Vadillo analysis, have been included in our updated meta-analysis.

Finally, Shanks & Vadillo argued that the selection criteria for the field studies in our original sub-analysis were not objective and hence biased. They do not mention that our finding was *contrary* to our hypothesis regarding the magnitude of field versus laboratory experiments. It is implausible that researchers selectively choose or omit their own experiments to contradict their hypothesis.

In the present updated sub-analysis, where we included the field experiments (i.e. [25], experiment 1; [24], experiment 3; [26]) that were not included in the Chen *et al*. [7] analysis, the *d*, as reported above, is 0.44. It would appear that despite the arguments of Shanks & Vadillo concerning one sub-analysis, namely of field versus laboratory experiments on goal priming, and given the exact and conceptual replications reported in Chen *et al*.'s [7] enumerative review, their arguments, to paraphrase Shakespeare, appear to be little more than a tempest in a teapot, especially in light of the effect size obtained by Chen *et al*. [7] regarding field versus laboratory goal priming experiments versus that obtained by Shanks & Vadillo [6], respectively. The one finding of utmost importance in this series of papers (i.e. [6,7,12]) is that Shanks & Vadillo [6], ardent critics of meta-analyses of priming experiments, based on their own literature review, reproduced our overall finding of a primed goal-organizational behaviour relationship.

## 5. Future research

The current meta-analysis is an update to Chen *et al*. [7] and included all available experiments of goal priming effects on outcomes relevant to organizational behaviour. We did not limit our search to only those experiments that reported particular effects, nor did we arbitrarily or prematurely discontinue our search when sufficient power had been realized. The results reported in the current study replicate those reported in Chen *et al*.

Given the novelty and experimental nature of this area of research in organizational psychology, we acknowledge several potential limitations as well as opportunities for future research. Several of the laboratory and field experiments in our dataset, for example, report small sample sizes. As such, field experiments on goal priming are at risk of being underpowered. With small samples, outliers in underlying studies could exaggerate reported results. In our dataset, however, observed effects in field experiments were consistent and robust. Only three field experiments excluded outliers from their respective data analyses, a number too small to determine whether outliers affected the results we have reported. Although outliers and small sample sizes could be problematic in primary studies, we anticipate that the use of meta-analysis accounts for these factors in estimations of overall effects [17]. That said, future field experiments should attempt where possible to use large samples, and account for the impact of outliers on reported effects.

In our comprehensive search of the goal priming literature, we found only one unpublished experiment [35] that fit our search criteria. Future meta-analyses and enumerative reviews of the literature should attempt to include doctoral dissertations and unpublished studies so as to minimize any potential selection bias, publication bias or under-reporting in the goal priming literature. For example, with regard to under-reporting Rauch & Frese [38] found that publication biases led to lower correlations between personality and entrepreneurial career choices in published versus unpublished papers. This was the result of entrepreneurship researchers' biases against personality measures.

In the original Chen *et al*. study, as well as in the present updated meta-analysis, we examined moderators that reflect study characteristics (e.g. modality and time lag) and those that provide a test of components of goal setting theory ([10]; e.g. goal specificity). Future experiments conducted in laboratory or field settings should broaden the examination of boundary conditions of primed goal effects to include characteristics of the setting, the persistence or repetition of the prime, and the extent to which the prime is dynamic, such as a video cue, or a still, such as an image or a photograph.

## Data Availability

The datasets supporting this article have been uploaded as part of the electronic supplementary material. The data are provided in the electronic supplementary material [39].

## Footnotes

1 We thank Dana Joseph for the analysis of our data.

2 ‘These methods (psychometric meta-analyses) correct not only for sampling error (an unsystematic artifact) but also for other systematic artifacts, such as measurement error, range restriction or enhancement, dichotomization of measures, and so forth’ [17, p. 339]. Moreover, the worry that small datasets from goal priming laboratory experiments may be the result of a researcher stopping as soon as a significant *p*-level has been obtained is an unlikely occurrence in field experiments. It is unlikely that a researcher would go to the human resources department and say ‘I want the data now to see whether the results are statistically significant. If they are not, I want to continue the study’.

3 Shanks & Vadillo [6] questioned the log transformation of the data collected for Shantz & Latham's [20] main field experiment. They failed to report that we conducted an exact replication of those results in two organizations where a log transformation of the data was not performed [22].

4 In addition to providing a conceptual replication of the second experiment conducted by Shantz & Latham [20], Lenoir & Matthews' [27] findings increase the external validity of Bargh's [28,29] automaticity model by showing that a goal that is primed has a positive effect on the behaviour of unpaid volunteers.

5 We made corrections to our original dataset for the Chen *et al.* [7] data analyses on 2 June 2020. We completed the current literature search on 28 November 2021.
